# Bone Marrow Soluble Mediator Signatures of Patients With Philadelphia Chromosome-Negative Myeloproliferative Neoplasms

**DOI:** 10.3389/fonc.2021.665037

**Published:** 2021-05-18

**Authors:** Juçara Gastaldi Cominal, Maira da Costa Cacemiro, Maria Gabriela Berzoti-Coelho, Illy Enne Gomes Pereira, Fabiani Gai Frantz, Elizabeth Xisto Souto, Dimas Tadeu Covas, Lorena Lobo de Figueiredo-Pontes, Maria Carolina Oliveira, Kelen Cristina Ribeiro Malmegrim, Fabíola Attié de Castro

**Affiliations:** ^1^ Department of Clinical Analyses, Toxicology and Food Science, School of Pharmaceutical Sciences of Ribeirão Preto, University of São Paulo, Ribeirão Preto, Brazil; ^2^ Center for Cell-Based Therapy, Regional Blood Center of Ribeirão Preto Medical School, University of São Paulo, Ribeirão Preto, Brazil; ^3^ Department of Clinical Hematology, Euryclides de Jesus Zerbini Transplant Hospital, São Paulo, Brazil; ^4^ Division of Hematology, Hemotherapy and Cellular Therapy, Department of Medical Imaging, Hematology, and Clinical Oncology, Ribeirão Preto Medical School, University of São Paulo, Ribeirão Preto, Brazil; ^5^ Division of Rheumatology, Allergy and Immunotherapy, Department of Internal Medicine, Ribeirão Preto Medical School, University of São Paulo, Ribeirão Preto, Brazil

**Keywords:** myeloproliferative neoplasms, soluble mediators, inflammation, angiogenesis, cytokines, bone marrow niche

## Abstract

**Background:**

Essential thrombocythemia (ET), polycythemia vera (PV), and primary myelofibrosis (PMF) are clonal hematological diseases classified as Philadelphia chromosome-negative myeloproliferative neoplasms (MPN). MPN pathogenesis is associated with the presence of somatic driver mutations, bone marrow (BM) niche alterations, and tumor inflammatory status. The relevance of soluble mediators in the pathogenesis of MPN led us to analyze the levels of cytokines, chemokines, and growth factors related to inflammation, angiogenesis and hematopoiesis regulation in the BM niche of MPN patients.

**Methods:**

Soluble mediator levels in BM plasma samples from 17 healthy subjects, 28 ET, 19 PV, and 16 PMF patients were determined using a multiplex assay. Soluble mediator signatures were created from categorical analyses of high mediator producers. Soluble mediator connections and the correlation between plasma levels and clinic-laboratory parameters were also analyzed.

**Results:**

The soluble mediator signatures of the BM niche of PV patients revealed a highly inflammatory and pro-angiogenic milieu, with increased levels of chemokines (CCL2, CCL5, CXCL8, CXCL12, CXCL10), and growth factors (GM-CSF M-CSF, HGF, IFN-γ, IL-1β, IL-6Ra, IL-12, IL-17, IL-18, TNF-α, VEGF, and VEGF-R2). ET and PMF patients presented intermediate inflammatory and pro-angiogenic profiles. Deregulation of soluble mediators was associated with some clinic-laboratory parameters of MPN patients, including vascular events, treatment *status*, risk stratification of disease, hemoglobin concentration, hematocrit, and red blood cell count.

**Conclusions:**

Each MPN subtype exhibits a distinct soluble mediator signature. Deregulated production of BM soluble mediators may contribute to MPN pathogenesis and BM niche modification, provides pro-tumor stimuli, and is a potential target for future therapies.

## Introduction

Essential thrombocythemia (ET), polycythemia vera (PV), and primary myelofibrosis (PMF) are classical myeloproliferative neoplasms (MPN) also known as Philadelphia chromosome (Ph)-negative MPN. These clonal diseases are characterized by single or multilineage hyperproliferation of the bone marrow (BM) that results in spontaneous accumulation of mature myeloid cells in the BM and peripheral blood. The erythroid lineage is the mostly affected in PV patients, while the megakaryocytic lineage from ET and PMF patients exhibit hyperplasia and atypia, and BM fibrosis. PMF patients also have increased or decreased number of granulocytes, monocytosis, and erythroid dysplasia (1, 2).

Disease pathogenesis is partially attributed to the presence of acquired driver mutations in Janus Kinase 2 (JAK2), calreticulin (CALR) or myeloproliferative leukemia virus (MPL) genes. These somatic mutations lead to abnormal activation of the JAK pathway, resulting in constitutive activation of their downstream effectors, specially STATs (2, 3). JAK2V617F is the most frequent driver mutation in MPN patients: it is detected in more than 90% of PV patients, and in about 50–60% of ET and PMF patients (4, 5). The CALR mutation is found in about 20–30% of ET and PMF patients, and is the second most frequent MPN-mutation (4, 5). Triple negative and MPL mutation are present in less than 12% of ET and PMF patients (4).

The crosstalk between inflammation and neoplastic cells plays a crucial role in disease development and progression. The BM of MPN patients is rich in inflammatory cytokines and growth factors, which form a pro-tumorigenic microenvironment that supports neoplastic cells and favors specific clinical phenotypes (6–8).

Cytokines and chemokines are key mediators of the immune system that regulate many complex signaling processes, and whose levels reflect the systemic and local immune activation status (9, 10). The co-participation of cytokines may result in activation or inactivation of immune pathways, and one cytokine can be secreted by different cell types in the same environment (10). In addition, cytokines act as important regulatory signals of hematopoiesis by inducing proliferation and/or survival of hematopoietic stem-cells (8, 11). Cytokines can also play a role as extrinsic factors that contribute to BM pathological changes (6, 7).

MPN are considered tumor inflammatory diseases. Over the past years, several studies have investigated how chronic inflammation contributes to MPN pathogenesis (6, 7, 12). We have recently described an altered cytokine profile in peripheral blood of MPN patients (13). PMF patients exhibit high inflammatory profile due overproduction of multiple pro-inflammatory cytokines and chemokines (13, 14); in these patients, the presence of JAK2V617F mutation is associated with high CXCL10 levels (13).

Considering the relevance of cytokines, chemokines, and growth factors (hereafter referred to as soluble mediators) as mediators of inflammation, angiogenesis and hematopoiesis regulation, here we examined the soluble mediator signature in the BM niche of ET, PV, and PMF patients. We also analyzed the correlation between cytokine levels and clinic-laboratory parameters.

## Materials and Methods

### Patients and Samples

The Ethics Committees for Human Research from the School of Pharmaceutical Sciences of Ribeirão Preto, from the University Hospital of the Ribeirão Preto Medical School (HC-FMRP; Ribeirão Preto, Brazil), and from the Euryclides de Jesus Zerbini Transplant Hospital (São Paulo, Brazil) approved the study protocol.

The studied groups consisted of 17 healthy volunteers (CTRL group) and 63 MPN patients (28 ET, 19 PV, and 16 PMF patients). The patients were recruited at the Bone Marrow Transplantation Unit of HCFMRP-USP and at the Euryclides de Jesus Zerbini Transplant Hospital. All MPN patients were diagnosed according to the 2016 World Health Organization criteria (1). Healthy BM donors were recruited at the Bone Marrow Transplantation Unit of HCFMRP-USP.

BM aspirates were collected from the left posterior iliac crest into EDTA tubes at the time of diagnosis. Plasma was separated from the BM samples by centrifugation at 400×*g* for 10 min at 4°C (Eppendorf 5810R centrifuge), and aliquots were stored at −80°C for further cytokine analysis.

### Risk-Stratification of MPN Patients

PV patients were classified into two risk categories: high and low. High-risk patients were the ones with age >60 years and/or history of vascular complications (including previous thrombosis, cardiovascular events and/or strokes). The patients who did not present the abovementioned two risk factors were classified as low risk (15).

The revised IPSET-thrombosis (r-IPSET-t) risk score splits ET patients into four risk categories: very low (age <60 years and absence of JAK2 mutation), low (age <60 years and presence of JAK2 mutation), intermediate (age >60 years and absence of JAK2 mutation), and high risk (age >60 years and presence of JAK2 mutation or vascular complication) (16).

The DIPSS-Plus scoring system was used to classify PMF patients into four categories: low, intermediate-1, intermediate-2, and high risk. The prognosis score considered the following risk factors: age, white blood cell and platelet counts, peripheral blood blast percentage, hemoglobin concentration, transfusion dependency, presence of constitutional symptoms, and unfavorable karyotype (17).

### Multiplex Assays

Soluble mediator levels were determined using a customized microbeads multiplex assay (Human Magnetic Luminex^®^ Assay, R&D Systems) performed on the Luminex1 MAGPIX1 System (Luminex Corporation). The cytokines and chemokines measured were interleukins (IL) IL-6, IL-1β, IL-12p70, IL-10, IL-17a, and IL-18; interferon gamma (IFN-γ); tumor necrosis factor alpha (TNF-α); IL-6 receptor subunit alpha (IL-6Ra); C–X–C motif ligands (CXC) CXCL8 (also known as IL-8), CXCL12 (also known as SDF-1, stromal cell-derived factor 1), CXCL10 (also known as IP-10, interferon-induced protein 10); and C–C motif chemokine ligands (CCL) CCL2 (known as MCP-1, monocyte chemotactic protein 1) and CCL5 (known as RANTES). The growth factors granulocyte-macrophage colony stimulating factor (GM-CSF), macrophage colony stimulating factor (M-CSF), granulocyte colony stimulating factor (G-CSF), hepatocyte growth factor (HGF), vascular endothelial growth factor (VEGF), and VEGF receptor 2 (VEGF-R2) were also quantified. Data were analyzed using the Milliplex Analyst software v3.5 (Millipore; VigeneTech Ind).

### Data Analyses

Mann–Whitney test was applied to compare differences in distribution of soluble mediators among MPN groups (ET, PV, and PMF) and patients’ JAK2V617F status. Spearman test was used for the correlation analysis of hematological parameters. GraphPad Prism 6.0 software (GraphPad Software) was used for statistical analysis. Significance was set at 0.05.

Cytoscape 3.7.2 software (available at htpp://cytoscape.org, National Institute of General Medical Sciences of the National Institutes of Health, USA) was used to construct the soluble mediator signatures (18), using the *r*-values (correlation coefficient) from the Spearman test that had *p*-value <0.05.

Overall soluble mediator profile was obtained by characterizing the general cytokine pattern of each group (18, 19). Each individual was classified as high or low mediator-producer, based on overall median values, as described previously by Vitelli-Aguiar et al. (19). The complete protocol of overall analyses was adapted from our previous work (13).

## Results

### Demographic Data and Clinic-Laboratory Profile of Study Cohort

The median age of MPN patients was 65.5 years (20–85), distributed as 63 (31–78), 62 (20–85), and 68.5 (54–80) years for ET, PV, and PMF patients, respectively. The median age of CTRL subjects was 49 (19–83) years. Male–female proportions in the studied CTRL, ET, PV, and PMF groups were 6–11, 5–23, 12–7, and 12–4, respectively. Demographic and clinic-laboratory characteristics of the MPN cohort, including age, gender, mutation *status*, risk stratification, fibrosis rate, transfusion dependency, treatment *status*, hematological parameters, and reticulin rate are summarized in **Table 1**. The individual characteristics of CTRL volunteers and MPN patients are summarized in **Tables S1**–**S4**.

**Table 1:** Demographic data and clinic-laboratory parameters from polycythemia vera (PV), essential thrombocythemia (ET), and primary myelofibrosis (PMF) patients.

Data	PV (n=19)	ET (n=28)	PMF (n=16)
**Age (years/range)**	62 (20–85)	63 (31–78)	68.5 (54–80)
**Gender (male %)**	12 (63.16)	5 (17.86)	12 (75)
**Mutation *status***			
*JAK2V617F^+^* (%)	19 (100)	13 (46.43)	9 (56.25)
*CALR^+^* (%)	0 (0)	7 (25)	5 (31.25)
*JAK2V617F^-^* (%)	0 (0)	5 (17.86)	0 (0)
*Double negative* (%)	0 (0)	3 (10.71)	2 (12.5)
**Treatment, n (%)**	4 (21.06)	10 (35.71)	5 (31.25)
*ASA*	2 (10.53)	2 (20)	0 (0)
*HU*	2 (10.53)	6 (60)	3 (60)
*ASA + HU*	0 (0)	2 (20)	0 (0)
*Anagrelide + ASA*	0 (0)	0 (0)	1 (20)
*Ruxolitinib*	0 (0)	0 (0)	1 (20)
**Vascular event, n (%)**	7 (36.84)	5 (17.86)	5 (31.25)
*NA*	0 (0)	4 (14.29)	0 (0)
**Transfusion dependency, n (%)**	0(0)	2 (7.14)	5 (31.25)
*NA*	1 (5.26)	3 (10.71)	0 (0)
**Fibrosis rate n, (%)**			
*0*	10 (52.64)	20 (71.44)	1 (6.25)
*1*	5 (26.32)	3 (10.71)	0 (0)
*2*	1 (5.26)	2 (7.14)	3 (18.75)
*3*	1 (5.26)	1 (3.57)	8 (50)
*4*	1 (5.26)	0 (0)	4 (25)
*NA*	1 (5.26)	2 (7.14)	0 (0)
**Reticulin rate n, (%)**			
*0*	0 (0)	14 (50)	0 (0)
*1*	0 (0)	4 (14.29)	0 (0)
*2*	0 (0)	1 (3.57)	4 (25)
*3*	0 (0)	1 (3.57)	8 (50)
*4*	0 (0)	1 (3.57)	4 (25)
*NA*	19 (100)	7 (25)	0 (0)
**Hematological parameters**			
*WBC count, ×10^3^/mm³ (range)*	12.1 (3.59–21)	7.28 (3.35–15.6)	5.85 (1.46–15.3)
*RBC count, ×10^6^/mm³ (range)*	6.33 (3.09–7.46)	4.34 (2.98–5.19)	3.66 (2.49–6.01)
*Hemoglobin, g/dl (range)*	16.3 (11.1–21.5)	13.2 (10.1–22.4)	11.1 (8.62–17.4)
*Hematocrit, % (range)*	49.1 (33.7–62)	40.65 (34.6–64.7)	35.15 (26.3–55.3)
*PLT count, ×10^3^/mm³ (range)*	605 (161–1502)	665.5 (304–1293)	305 (71.7–917)

ASA, acetylsalicylic acid; CALR^+^, positive for calreticulin mutation; Double negative, negative for JAK2V617F and CALR mutation; HU, hydroxycarbamide; JAK2V617F^+^, positive for JAK2V617F mutation; JAK2V617F^−^, negative for JAK2V617F mutation; NA, data not available; PLT, platelets; RBC, red blood cells; VE, previous vascular event; WBC, white blood cells.

### Soluble Mediator Levels in the BM Niche of PV, ET and PMF Patients

**Figure 1:**
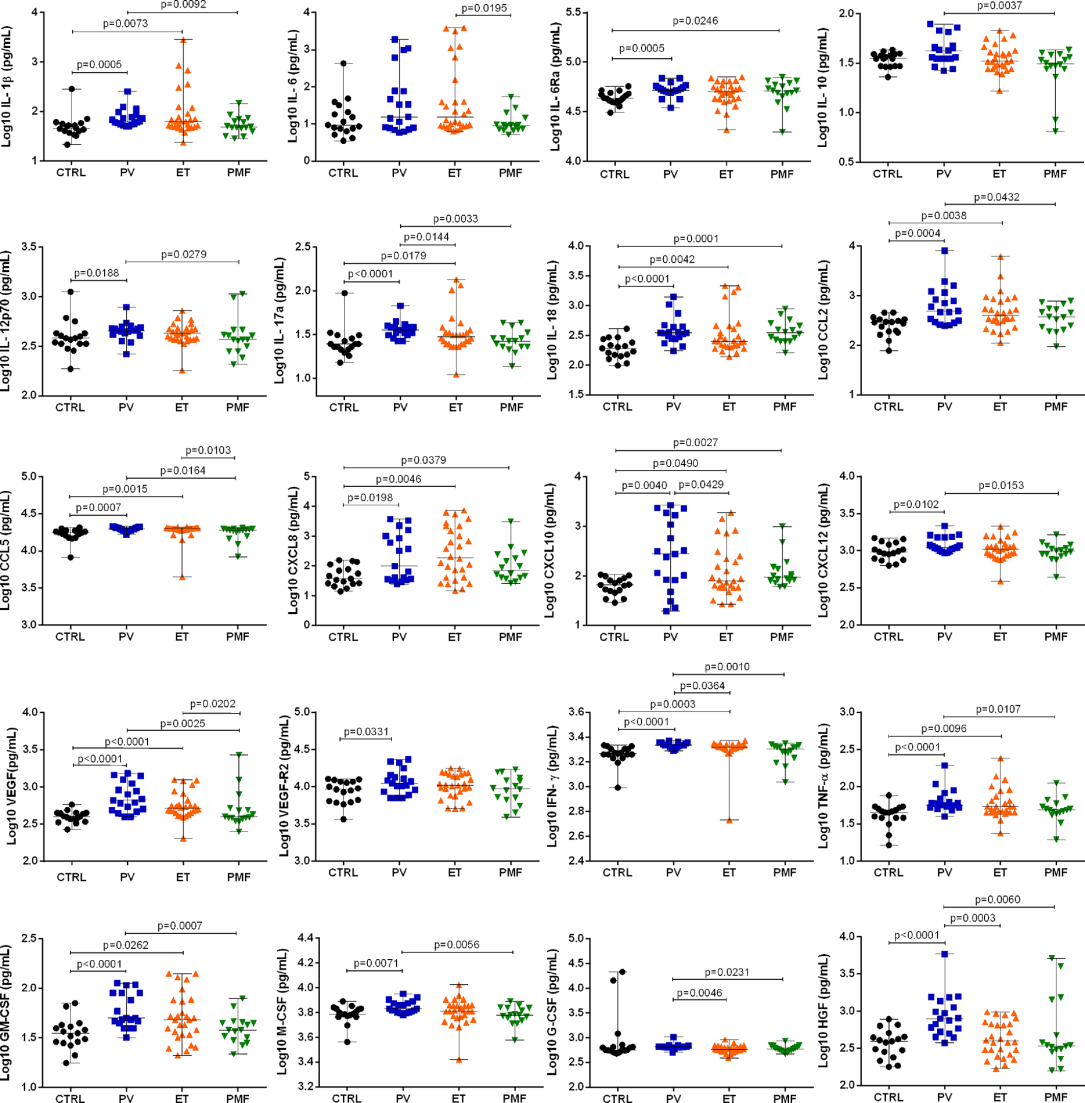
Bone marrow plasma levels of inflammatory soluble mediators in healthy subjects (CTRL; n = 17) and patients with essential thrombocythemia (ET, n = 28), polycythemia vera (PV, n = 19), and primary myelofibrosis (PMF, n = 16). The concentration of all the soluble mediators was determined using a Multiplex assay. Significant differences when p <0.05, Mann–Whitney test.

Compared with CTRL, the BM niche of PV patients presented increased levels of inflammatory cytokines and angiogenesis- and hematopoiesis-related factors, including CCL2, CCL5, CXCL8, CXCL10, CXCL12, GM-CSF, HGF, IFN-γ, IL-1β, IL-6Ra, IL-12p70, IL-17a, IL-18, M-CSF, TNF-α, VEGF, and VEGF-R2 (**Figure 1** and **Table S5**).

ET patients exhibited augmented levels of CCL2, CCL5, CXCL8, CXCL10, GM-CSF, IFN-γ, IL-1β, IL-17a, IL-18, TNF-α, and VEGF, when compared with CTRL. MF patients displayed higher levels of only CXCL8, CXCL10, IL-6Ra, and IL-18, as compared with CTRL (**Figure 1** and **Table S5**).

All MPN categories presented high production of the chemokines IL-18, CXCL10 and CXCL8. Compared with PV patients, ET patients had lower levels of G-CSF, HGF, IFN-γ, IL-10, and IL-17a in the BM niche, while PMF patients presented lower levels of CCL2, CCL5, CXCL12, G-CSF, GM-CSF, HGF, IFN-γ, IL-1β, IL-10, IL-17a, IL-12p70, M-CSF, TNF-α, and VEGF. The BM niche of ET patients exhibited higher levels of CCL5, IL-6 and VEGF than PMF patients.

The present results indicated that the BM levels of IL-17, IFN-γ, G-CSF, and HGF in PV patients were higher than those detected in PMF and ET patients. Hence, PV patients seem those with more unique soluble mediators profile as compared to the other MPN.

### Categorical Analyses of Soluble Mediator Production in BM Niche of MPN Subtypes

Categorical analyses were performed to better comprehend the soluble mediator production patterns and the differences among MPN subtypes. Patients were stratified into high and low producers of soluble mediators using the overall median as cut-off point. High producer was the individual whose soluble mediator production value was higher than the overall median, while low producer was the individual whose soluble mediator production level was equal to or lower than the overall median. The frequency of high producers of each mediator was calculated for all disease and CTRL subsets. The production of each mediator was considered relevant when the frequency of high producers exceeded 50% (13, 19, 20).

The CTRL group did not produce any of the mediators in relevant amounts, since less than 50% of the individuals were high producers (**Figure S1**). Soluble mediator production in BM niche differed among MPN subtypes. PV patients exhibited remarkably dysregulated production of soluble mediators, with relevant production of the 20 mediators analyzed. Only PV group showed high producers for IL-10, CXCL12, IFN-γ, G-CSF and HGF in a relevant frequency. Differently from PMF, PV and ET group showed relevant production of IL-6, IL-1β, IL-17a, IL-12p70, CCL5, VEGF, VEGF-R2, TNF-α, GM-CSF and M-CSF.

ET patients produced most of the mediators in relevant amounts, but PMF patients exhibited relevant production of only five mediators (**Figure S1**), among them IL-6Ra, IL-18 and CXCL10 were present in PMF but not in ET patients. Interestingly, all MPN categories presented high production of the chemokines CXCL8 and CCL2 (>50% high producers).

Spider charts summarize the soluble mediator signatures and enable visual comparison among the CTRL, ET, PMF, and PV groups (**Figure S2**).

### Soluble Mediator Networks in MPN Subtypes

**Figure 2:**
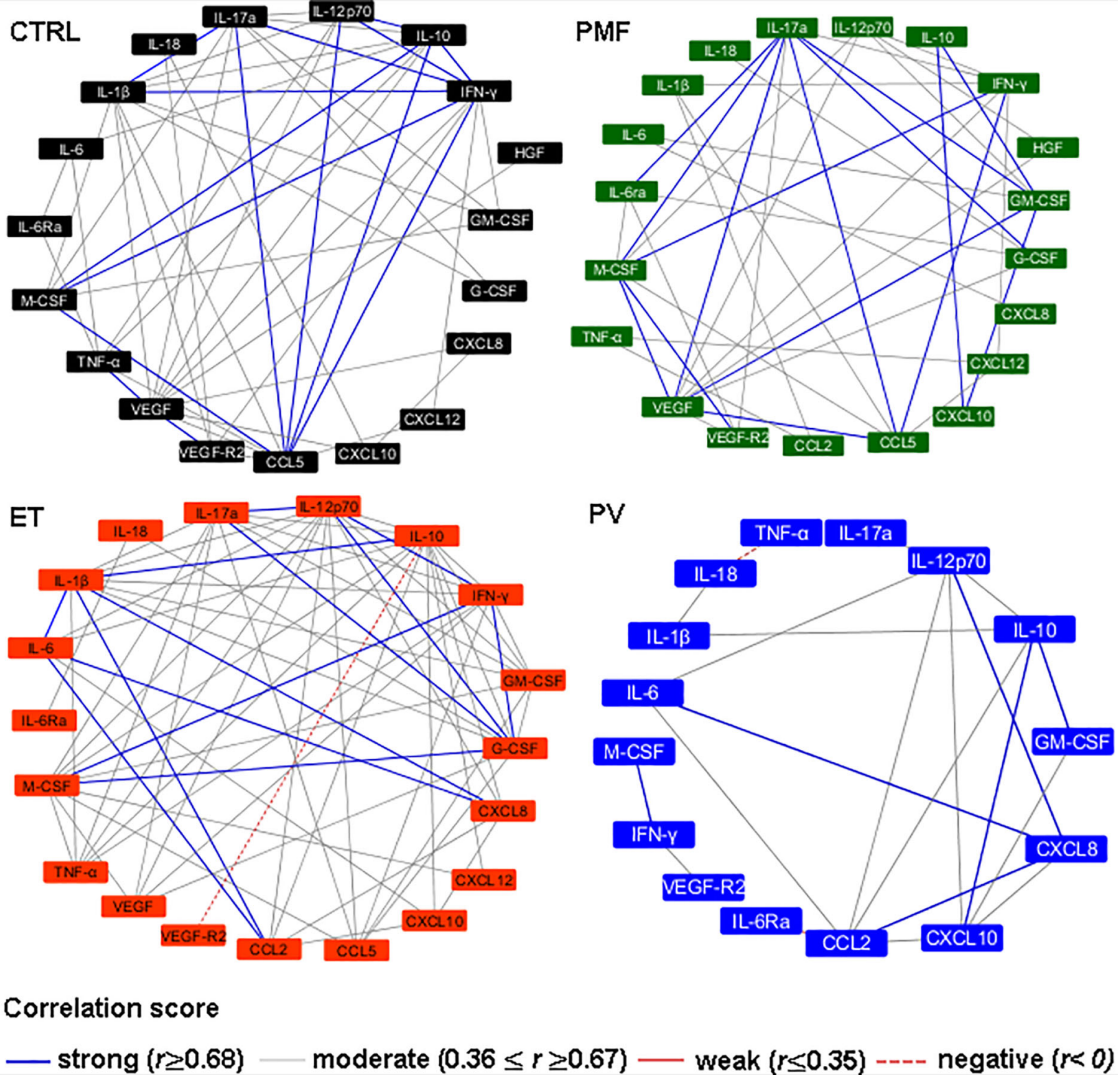
Soluble mediators interaction networks in healthy subjects (CTRL, n = 17) and patients with essential thrombocythemia (ET, n = 28), polycythemia vera (PV, n = 19), and primary myelofibrosis (PMF, n = 16). The correlations were stratified according to *r*-values: strong (blue lines, *r* ≥0.68), moderate (gray lines, 0.36 ≤ *r* ≥ 0.67) and negative (dashed red line, *r* <0). The correlations depicted in this figure were significant (p <0.05).

After identifying the soluble mediator signature for each disease (ET, PV, and PMF) and CTRL group, we analyzed the existence of correlation between the mediator levels (**Figure 2**). Correlations were stratified into negative (*r <*0), weak (*r ≤*0.35), moderate (*r* ≥0.36 and *r ≤*0.67) or strong (*r* ≥0.68). The CTRL group exhibited the highest number of strong correlations, and similar findings were obtained in ET patients (IL-1β with IL-17a and IFN-γ; IL-10 with IL-12p70, IFN-γ and M-CSF; IL-17a with CCL5 and IFN-γ; IL-12p70 with CCL5 and IFN-γ; CCL5 with IFN-γ and M-CSF; VEGF with VEGF-R2; and IFN-γ with M-CSF). PMF patients also showed many strong interactions (between IL-17a with IL-6Ra, CCL5, VEGF, GM-CSF, G-CSF and M-CSF; IL-10 with CXCL10 and GM-CSF; CCL5 with IFN-γ and VEGF; CXCL10 with GM-CSF; M-CSF with IFN-γ and VEGF-R2), while PV patients were highly different from the CTRL group and showed the lowest number of strong correlations (CXCL8 with IL-6, IL-12p70 and CCL2; IL-10 with GM-CSF and CXCL10; and M-CSF with IFN-γ). These results highlighted the deregulation of soluble mediator network in PV patients versus other MPN categories and CTRL group.

### Correlation Between the BM Soluble Mediator Levels and Patients’ Clinic-Laboratory Parameters

**Figure 3:**
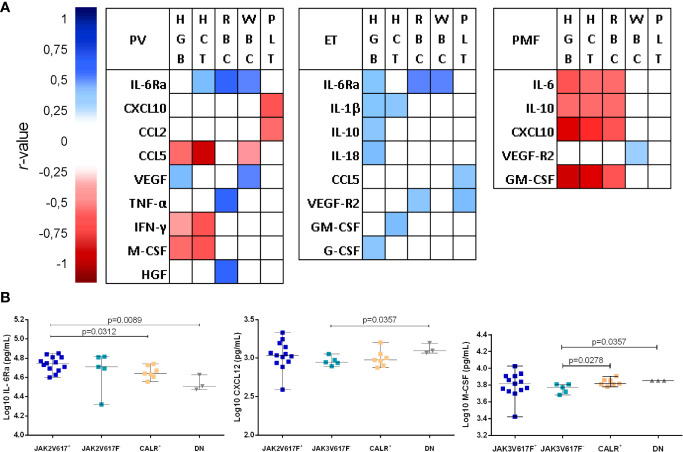
**(A)** Correlation between soluble mediator levels and laboratory parameters of healthy subjects (CTRL, n = 17) and patients with essential thrombocythemia (ET, n = 28), polycythemia vera (PV, n = 19), and primary myelofibrosis (PMF, n = 16). The Spearman coefficient (*r*-value) was represented by a color gradient that ranged from close to 1 (dark blue) to −1 (dark red); white indicates no correlation. The correlations depicted in this figure were significant when p <0.05. HCT, hematocrit; HGB, hemoglobin concentration; PLT, platelet count; RBC, red blood cell count; WBC, white blood cell count. **(B)** Association between driver mutation *status* and IL-6Ra, CXCL12, and M-CSF levels in bone marrow plasma from patients with ET. JAK2V617^+^ (n = 13 ET patients positive for JAK2V617F mutation). JAK2V617F^-^ (n = 5; ET patients negative for JAK2V617F mutation). CALR^+^ (n = 7; patients positive for calreticulin mutation). DN (n = 3; double negative for CALR and JAK2V617 patients). Statistical difference when p <0.05, Mann–Whitney test.

We analyzed the potential correlation between soluble mediator levels and the major clinic-laboratory parameters (**Figure 3A**). The patients’ hematological parameters analyzed were hemoglobin concentration, hematocrit, and white blood cell, red blood cell, and platelet counts.

The following correlations among soluble mediators and MPN clinical parameters were found:

In PV, hemoglobin concentration positively correlated with VEGF and HGF, and negatively with CCL5 and IFN-γ; red blood cell count positively correlated with IL-6Ra and TNF-α; hematocrit positively correlated with IL-6Ra, and negatively correlated with CCL5 and IFN-γ; white blood cell count positively correlated with IL-6Ra and VEGF, and negatively with CCL5; and platelet counts negatively correlated with CXCL10 and CCL2 (**Table S6**).

ET patients displayed positive correlations between hemoglobin concentration with IL-6Ra, IL-1β, IL-10, IL-18 and G-CSF; red blood cell count with IL-6Ra and VEGF-R2; hematocrit with GM-CSF and IL-1β; white blood cell count with IL-6Ra; and platelet count with VEGF-R2 and CCL5 (**Table S7**).

In PMF, white blood cell count positively correlated with VEGF-R2. Negative correlations among hemoglobin concentration, red blood cell count and hematocrit with IL-6, IL-10, CXCL10 and GM-CSF were also observed (**Table S8**). No similar patterns of correlation between soluble mediator levels and the clinic-laboratory parameter were obtained in PV, ET and PMF.

**Figure 4:**
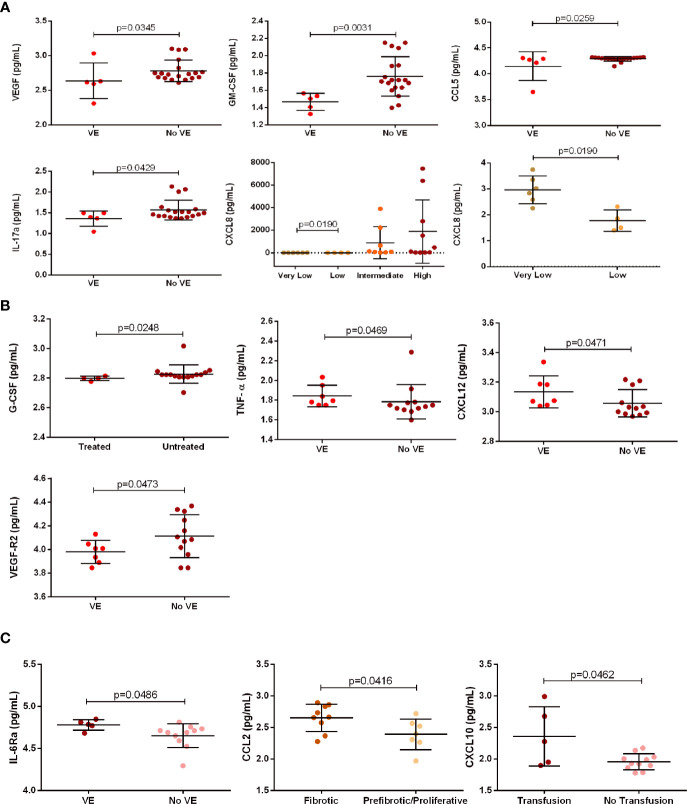
Association between clinical parameters of patients with essential thrombocythemia (ET, n = 28), polycythemia vera (PV, n = 19), and primary myelofibrosis (PMF, n = 16) and soluble mediator levels. **(A)** ET patients: association between VEGF, GM-CSF, CCL5, and IL-17 levels and the presence (n = 5) or absence (n = 19) of VE; and CXCL8 levels and risk-stratification in very low (n = 6), low (n = 4), intermediate (n = 8) and high (n = 10) risk. **(B)** PV patients: association between G-CSF levels and administration (n = 4) or not (n = 15) of drug treatment; and association between TNF-α, CXCL12, and VEGF-R2 levels and the presence (n = 7) or absence (n = 12) of VE. **(C)** PMF patients: association between IL-6Ra levels and the presence (n = 5) or absence (n = 11) of VE; association between CCL2 levels and fibrotic disease (n = 9) or pre-fibrotic/proliferative (n = 7) stage; and association between CXCL10 levels and transfusion dependency (n = 5) or no transfusion necessity (n = 11).

Risk-stratification analysis showed in very low risk ET patients (n = 6) higher CXCL8 levels than those detected in low risk ET patients (n = 4) (**Figure 4A**). No relationships were found among soluble mediators and PV and PMF risk *status*.

Regarding therapy, treated PV patients (with hydroxycarbamide or acetylsalicylic acid) had lower G-CSF levels than untreated PV patients (**Figure 4B**). There was no association between soluble mediator levels and treatment in ET and PMF patients

Five ET patients with vascular event (including thrombosis, cardiovascular events and/or strokes) exhibited lower levels of IL-17, CCL5, GM-CSF, and VEGF than ET patients with no vascular event (n = 17) (**Figure 4A**). PV patients with vascular event (n = 7) displayed higher levels of CXCL12, TNF-α and VEGF-R2 than PV patients with no vascular event (n = 12) (**Figure 4B**). PMF patients with vascular event (n = 5) had increased levels of IL-6Rα when compared with patients with no vascular event (n = 11) (**Figure 4C**).

Finally, we analyzed, in PMF patients, the potential association between soluble mediator levels and transfusion dependency, and disease stages (**Figure 4C**). PMF patients with transfusion dependency had higher CXCL10 levels than those with no dependency. In addition, PMF patients in pre-fibrotic/proliferative stage had elevated CCL2 levels when compared with those in fibrotic stage.

### JAK2V617F^+^ ET Patients Have High IL-6Ra Levels

The soluble mediators levels of ET and PMF patients were stratified according to their driver mutation *status*. ET JAK2V617F^+^ showed higher IL-6Ra levels than those with CALR mutation, and double negative (DN) for JAK2V617F and CALR. ET CALR mutated patients displayed elevated M-CSF levels than DN; and JAK2V617F^-^ patients showed lower levels of M-CSF and CXCL12 than DN patients (**Figure 3B**). There was no association between soluble mediators levels and mutation *status* in PMF patients.

The extent of production (i.e. high versus low producers) of soluble mediators according to the presence of driver mutations was also analyzed in ET (**Figure S3**) and PMF (**Figure S4**) patients. JAK2V617F^+^ ET patients were high producers of CCL2, CXCL10, CXCL12, IL-1β, IL-6Ra, IL-18, and TNF-α; while CALR mutated were high producers of GM-CSF, G-CSF, HGF, IFN-γ, IL-12p70, and IL-17a. The soluble mediators were differentially produced in PMF subgroups, JAK2V617F^+^ patients were high producers of CXCL8, and low producers of G-CSF, M-CSF, and VEGF-R2, while CALR mutated patients showed the opposite profile of these mediators’ production.

## Discussion

Both the MPN clone and BM-resident cells maintain the cytokine-mediated inflammatory microenvironment in a feedback loop mechanism that also maintains a pro-tumorigenic environment. Neoplastic cells secrete pro-inflammatory and angiogenic mediators that promote autocrine and paracrine stimulation of fibroblasts, endothelial cells, and stromal cells. In contrast, mediators produced in the BM have the potential to modify the phenotype of resident cells, stimulate angiogenesis and fibrosis, and thereby influence neoplastic cell survival, proliferation, and progression (7, 21, 22).

Little is known about the cytokine milieu in the BM niche of MPN patients. Most of the studies have reported the presence of angiogenesis-related molecules by immunohistochemistry analysis and associated their high levels with the presence of neo-angiogenesis and fibrosis in BM (23, 24).

In physiological state, the control of cytokines and chemokines production include an intricate of regulatory mechanism, with an inhibitory feedback and synergic actions to guarantee the balance of mediators levels, non-inflammatory *status*, and tissue homeostasis (25, 26).

PV patients exhibited a unique soluble mediator signature, as demonstrated by the overall and single analysis of soluble mediators. PV patients had higher levels of IL-17, IFN-γ, G-CSF and HGF, as compared with PMF and ET patients. These cytokines may be a useful tool in differential diagnosis of MPN.

The increased soluble mediator levels in BM niche from MPN patients could be partially explained by chronic inflammation associated with oncogenesis (7, 21). Chronic inflammation may be linked to hypoxia due to cell accumulation in the BM, which in turn was associated with JAK/STAT pathway activation by IL-6, IL-11, VEGF, HGF, PDGF, and TGF-β that mediates cell survival and proliferation, and thereby contributes to MPN pathogenesis (27, 28). Cytokine overproduction could also result from cancer-associated genetic mutations (27). Most of our results demonstrate that soluble mediators are not influenced by driver mutation *status* and corroborate previous studies on MPN patients (13, 28–31).

It is worth to note that IL-6Ra and M-CSF levels, in this study, were associated with JAK2V617F and CALR driver mutations, respectively. JAK2V617F^+^ ET patients presented high BM IL-6Ra levels, while CALR^+^ ET patients exhibited high M-CSF levels. Categorical analysis of soluble mediator production according to driver mutation *status* identified that the group of CALR^+^ ET patients had the lowest number of high producers.

The trans-signaling of IL-6/IL-6R soluble receptor (sIL-6R) complex lead to subsequent activation of JAK/STAT, MAPK and PIK pathways, and it can be activated in all types of cells, including cells not responsive to IL-6 alone (32). An observational epidemiological study reported that polymorphisms that cause loss of IL-6R function are associated with reduced risk of JAK2V617F mutation and MPN (33).

M-CSF-stimulated human macrophages have growth-promoting and proangiogenic phenotype with tissue repair potential under conditions of induced inflammation (34). In the present study, CALR^+^ MPN patients had higher M-CSF levels than JAK2V617F^+^ patients. It is well-known that CALR^+^ patients have better prognosis and higher overall survival than JAK2V617F^+^ patients (35). Our results, combined with the abovementioned references (32–35), may suggest that: 1) high IL-6Ra levels favor JAK/STAT pathway activation and the oncoinflammatory state in ET patients; 2) high M-CSF levels favor tissue homeostasis and attenuate inflammation in BM from CALR^+^ patients.

The BM milieu and the peripheral blood systemic profile from MPN patients are distinct. Many authors have reported that MPN patients develop a robust and systemic inflammatory response in peripheral blood (13, 29, 36, 37). In our study, ET and PMF patients had mild BM niche inflammation, while PV patients exhibited the most prominent and diffuse inflammatory response in BM niche, among the studied MPN categories.

PMF is the MPN subtype with higher number of alterations in the BM niche, including the presence of fibrosis and defective hematopoiesis (1). Indeed, BM fibrosis seems to result from continuous and long-lasting shift of the cytokine milieu rather than a specific genetic trigger (38). In our study, the cytokine milieu in PMF patients was similar to the CTRL group, despite the increased levels of CXCL8, CXCL10, IL-18, and IL-6Ra. Indeed, we identified CCL2, CXCL8, CXCL10, and IL-18 as MPN-associated cytokines, due to their prominent levels in BM niche of all MPN subtypes.

IL-18 is considered an inflammasome product whose main function is to promote IFN-γ secretion (39). IL-18 secreted by BM stroma elicits the growth of leukemia blast cells and contributes to progression of T-cell acute leukemia (40). Elevated BM IL-18 levels are also associated with poor overall survival of multiple myeloma patients (41). IL-18 has been implicated in induction of fibrosis in idiopathic pulmonary fibrosis and heart inflammation; blockage of IL-18 activity has antifibrotic effects (42, 43). As described in other hematological malignancies and diseases characterized by accentuated fibrosis, we hypothesize that IL-18 may contribute to tumorigenesis and BM fibrosis process in MPN.

CXCL8 and CXCL10 are important to regulate hematopoietic stem cells (44, 45) and mediate inflammation-driven angiogenesis due to CXCL10 angiostatic activity and CXCL8 angiogenic properties (46). The contribution of CXCL8 to tumorigenesis has been described in patients with acute myeloid leukemia, in which high CXCL8 levels are secreted by BM mesenchymal stromal cells and support the proliferation and survival of leukemic cells (47). Blockage of CXCL8 expression in PMF CD34^+^ cells promotes cell proliferation and megakaryocyte differentiation (48). Neutralization of the CXCL8 receptors CXCR1 and CXCR2 enhances PMF megakaryocyte cell proliferation, indicating that CXCL8 and its receptor are involved in megakaryocyte abnormalities and contribute to PMF pathogenesis (48). The two last reports cited (47, 48) confirmed our findings and stressed the importance of these mediators in MPN subtypes, manly by regulating neoplastic cell proliferation, survival, and differentiation.

CXCL8 is a pre-fibrotic cytokine (21) whose levels are increased in BM biopsies of PMF patients, as demonstrated by immunohistochemistry. MF CD34^+^ CXCL8-secreting clones are associated with patients with high-grade reticulin fibrosis in BM (49). Moreover, elevated CCL2 levels in MPN patients are associated with fibrosis and poor prognosis (28, 29, 36, 37). The expression of inflammatory genes, especially *CCL2* and *CXCL10*, is upregulated in patients with overt fibrosis, indicating that pro-inflammatory gene upregulation is associated with BM fibrosis, independently of the MPN (30). CCL2 and CXCL8 also exert a myelosuppressive effect that can disturb normal hematopoiesis (50).

In summary, these reports corroborate our findings and reinforce the contribution of CCL2, CXCL8, CXCL10, and IL-18 for MPN pathogenesis by promoting hematopoietic niche modifications, activation of angiogenesis, and deregulation of hematopoiesis. Only CXCL8 has been previously reported as a MPN-associated cytokine (21, 22, 51); this could be explained by the distinct cytokine levels detected in peripheral blood and BM.

The network analysis of soluble mediators revealed a distinct integrative system among PV and the other studied groups (ET, PMF and Control). Most of the soluble mediators interaction found in our study presented biological relevance, and resides in their synergistic interactions, which could be observed between: 1) IL-1β and IL-12 inducing IFN-γ secretion (52); 2) IL-1β, TNF-α and IL-6 promoting VEGF secretion (53); 3) GM-CSF interaction with M-CSF/G-CSF resulting in increase of granulopoiesis and monocytopoiesis (54); 4) IFN-γ with IL-1β and TNF-α upregulates CCL5 expression (55). These correlations were observed in ET and PMF patients.

PV patients display very strong positive correlations only between a few cytokine and chemokine molecules (IL-6, CXCL-8, IL-12 and CCL2). This data suggests that the immune imbalance in BM microenvironment is more prominent in PV than in ET and PMF patients. Literature reported that these molecules are associated with a pro-inflammatory *status*, occurrence of vascular events and oncoinflammation.

We demonstrated that soluble mediator levels in the BM niche correlated to clinic-laboratory parameters, including hemoglobin concentration, hematocrit, and white blood cell, red blood cell, and platelet counts. Many studies corroborate our findings and have demonstrated cytokine-phenotype associations related to pro-inflammatory *status* in MPN patients’ serum (13, 36, 37). In PMF patients, CXCL8 is associated with leukocytosis, and CXCL10 levels correlate with thrombocytopenia (37). In PV patients, high levels of IL-12 are associated with hematocrit; high IL-1β and HGF levels are associated with leukocytosis; low IL-6 and FGF (fibroblast growth factor) levels are associated with hemoglobin concentration; and low GM-CSF levels are associated with thrombocytosis (36). In PMF patients, the elevation of CXCL10 was associated with thrombocytosis and decreased levels of CXCL10 and IL-17 with erythropenia, while in ET patients the low levels of TNF-α was associated with thrombocytosis (13). The different soluble mediator association patterns among MPN subtypes may explain their distinct clinical features.

In our study, soluble mediator levels were not significantly associated with disease prognosis risk, probably due to the small number of patients enrolled. The reduced soluble mediator levels in treated MPN patients suggest that drug treatment influenced the production of many soluble mediators, although most of the comparisons did not reach statistical significance—except for G-CSF levels in PV patients.

It is well-known that hydroxycarbamide (hydroxyurea), a cytoredutor drug indicated for MPN treatment, is capable of lowering serum inflammatory markers such as TNF-α, IL-6, CXCL8, and IL-1β in sickle cell disease patients (56, 57). This drug suppresses production of pro-inflammatory cytokines in monocytes from sickle cell anemia patients (57); however, its effect on cytokine levels of MPN patients is poorly studied. The anti-inflammatory action of hydroxycarbamide relies on the hematological remission resulting from myelosuppression, reduction of leukocyte counts (58), and the drug effects on monocytes, as pointed out in sickle cell anemia.

The frequency of vascular events was associated with different setups of soluble mediators among MPN subtypes. PV patients exhibited increased TNF-α, CXCL12, and VEGFR2 levels; ET patients displayed increased IL-17, CCL5, GM-CSF, and VEGF levels; and PMF patients had increased IL-6Ra levels. Elevated GM-CSF and IL-12 serum levels are associated with the lack of vascular complications in ET and PV patients; these cytokines may also help to select the treatment regimen (29). In addition, CCL5 levels are associated with microvascular manifestations in PV patients (36). Augmented levels of angiogenic cytokines as VEGF, soluble vascular endothelial growth factor receptors 1 and 2, and placenta growth factor, as well as the increased number of endothelial cells and endothelial precursors are associated with high risk of thrombotic events in ET and PV patients (59). The levels of coagulation activation markers did not differ with respect to the JAK2V617F mutational status, but the association between endothelial cells and leukocytes may contribute to thrombosis (59).

Activated platelets are the mainly secretors of CXCL12. Upregulated CXCL2 expression and secretion may favor the development of cardiovascular diseases, while the fast increase of CXCL12 in peripheral blood platelets can be used as biomarker for cardiac injury (60). Patients with ischemic stroke have increased TNF-α serum levels and higher risk for cardiovascular diseases, compared with healthy volunteers (61). The studies reported in the two last paragraphs (26, 33, 53, 54) corroborate our findings on the influence of soluble mediators on vascular events and support the concept that the inflammatory environment is a crucial stimulus for the initiation and development of thrombo-hemorrhagic events and cardiovascular diseases (7).

In PMF patients, there were associations between high CXCL10 levels and transfusion requirement, and high CCL2 levels and pre-fibrotic/proliferative disease stage. These results are supported by the association between upregulated *CXCL10* and *CCL2* gene expression and increased fibrosis in BM niche (30). Our findings revealed a potential utility of monitoring CCL2 levels during PMF course, and provide a new tool to measure BM fibrosis evolution.

## Conclusions

Taken together, our findings demonstrate the existence of different soluble mediator signatures for each MPN subtype, among which PV patients present the highest levels of inflammatory and angiogenic soluble mediators. We identified CXCL8, CXCL10, IL-18, and CCL2 as MPN-associated soluble mediators; IL-17, IFN-γ, and HGF as biomarkers for PV; and CCL2 as biomarker for monitoring the BM fibrosis. In addition, specific mediators are potential targets for developing future therapies to prevent BM transformation in MPN patients. The molecular mechanisms involved in cellular malignant transformation by the inflammatory/angiogenic BM milieu in MPN patients are currently unknown and further investigations are underway.

## Data Availability Statement

The original contributions presented in the study are included in the article/**Suppelementary Material**. Further inquiries can be directed to the corresponding author.

## Ethics Statement

The studies involving human participants were reviewed and approved by The Ethics Committee for Human Research of the School of Pharmaceutical Sciences of Ribeirão Preto, the University Hospital of the Ribeirão Preto Medical School, and the Euryclides de Jesus Zerbini Transplant Hospital. The patients/participants provided their written informed consent to participate in this study.

## Author Contributions

JC, KM, and FC conceived and designed the study. JC, MC, MB-C, IP, and FF prepared the material and collected and analyzed data. LF-P, ES, and MO selected and recruited the patients and performed their clinical evaluation. DC, FC, and KM acquired funding and provided the resources. JC and MC wrote the first draft of the manuscript, while FC, KM, and MO revised and edited it. All authors commented on previous versions of the manuscript. All authors contributed to the article and approved the submitted version.

## Funding

This study was supported in part by the Coordination for the Improvement of Higher Education Personnel (CAPES; Finance Code 001), by the São Paulo Research Foundation (FAPESP; Regular Research grant #2018/19714-7; CTC grant #2013/08135-2; INCTC grant #2014/50947-7; Young Investigator grant# 2015/21866-1), and by the National Council for Scientific and Technological Development (CNPq grants #163064/2018-0, #169093/2018-2, and #305959/2018-2). MC and MB-C are recipients from FAPESP scholarships (grants #2018/01756-5 and #2015/23555-3, respectively).

## Conflict of Interest

The authors declare that the research was conducted in the absence of any commercial or financial relationships that could be construed as a potential conflict of interest.
