# Supplementary material for: Bone Marrow Soluble Mediator Signatures of Patients With Philadelphia Chromosome-Negative Myeloproliferative Neoplasms
Source: Front Oncol. 2021 May 18;11:665037. doi: 10.3389/fonc.2021.665037 (PMC8167065; doi:10.3389/fonc.2021.665037)
Supplement: Supplementary file 1 [file DataSheet_1.pdf]

Supplementary Material

Table S1. Demographic data from healthy subjects (control group, CTRL)

Patient ID	Age (years)	Gender
C11	68	Female
C13	49	Female
C15	50	Male
C16	46	Female
C17	83	Female
C18	31	Female
C19	66	Female
C20	58	Male
C22	54	Female
C23	72	Male
C24	38	Female
C25	60	Female
C100	36	Male
C101	24	Female
C102	37	Male
C103	34	Female
C104	19	Male

Table S2. Demographic data and clinic-laboratory parameters from patients with polycythemia vera (PV).

Code	Mutation	Age	Gender	Treatment	Risk	VE	Fibrosis rate
P77	JAK2V617F$^+$	76	M	---	High	Yes	0
P83	JAK2V617F$^+$	75	M	HU	High	Yes	3
P86	JAK2V617F$^+$	57	M	---	High	Yes	2
P90	JAK2V617F$^+$	61	M	ASA	High	No	4
P101	JAK2V617F$^+$	69	F	---	High	No	1
P102	JAK2V617F$^+$	61	F	---	High	No	0
P105	JAK2V617F$^+$	78	F	---	High	No	0
P106	JAK2V617F$^+$	67	M	---	High	Yes	0
P108	JAK2V617F$^+$	65	M	ASA	High	No	NA
P71	JAK2V617F$^+$	54	F	---	Low	No	0
P85	JAK2V617F$^+$	60	M	---	High	No	0
P59	JAK2V617F$^+$	66	M	---	High	No	1
P43.2	JAK2V617F$^+$	71	F	---	High	No	0
P45.2	JAK2V617F$^+$	61	M	---	High	No	0
P51.2	JAK2V617F$^+$	62	M	---	High	Yes	0
P63.2	JAK2V617F$^+$	63	M	---	High	Yes	1
P87	JAK2V617F$^+$	51	F	---	Low	No	1
P47.2	JAK2V617F	31	F	---	Low	No	0
P80	JAK2V617F$^+$	46	M	HU	Low	Yes	1

ASA: acetylsalicylic acid. F: female. HU: hydroxycarbamide (hydroxyurea). JAK2V617F$^+$: positive for JAK2V617F mutation. M: male. NA: not available. VE: previous vascular event.

Table S3. Demographic data and clinic-laboratory parameters from patients with essential thrombocythemia (ET).

Code	Mutation *status*	Age	Gender	Treatment	Risk	VE	Fibrosis rate	Reticulin rate
P28	JAK2V617F$^+$	74	F	---	High	No	2	NA
P55	JAK2V617F$^+$	35	F	HU	High	Yes	0	0
P91	JAK2V617F$^+$	81	M	ASA, HU	High	No	3	3
P35.2	JAK2V617F$^+$	72	M	HU	High	No	2	0
P46.2	JAK2V617F$^+$	77	M	---	High	Yes	0	0
P49	JAK2V617F$^+$	76	F	---	High	NA	NA	NA
P58.2	JAK2V617F$^+$	69	F	---	High	Yes	0	0
P38.2	JAK2V617F$^+$	46	F	HU	High	Yes	0	1
63	JAK2V617F$^+$	52	F	ASA	Low	No	0	1
P68	JAK2V617F$^+$	66	F	HU	High	NA	1	4
P81	JAK2V617F$^+$	36	F	---	Low	NA	0	0
P93	JAK2V617F$^+$	59	F	---	Low	No	0	0
P104	JAK2V617F$^+$	42	F	---	Low	No	NA	NA
P12	CALR$^+$	66	F	HU	Int	No	0	0
P66	CALR$^+$	77	F	ASA	Int	No	1	NA
P76	CALR$^+$	76	F	---	Int	No	0	2
P78	CALR$^+$	20	F	---	Very low	No	0	0
P53	CALR$^+$	62	M	HU	Int	No	0	0
P54.2	CALR$^+$	59	F	---	Very low	No	0	NA
P56.2	CALR$^+$	85	F	---	Int	No	0	NA
P53.2	DN	51	F	---	Very Low	No	0	0
P75	DN	61	F	---	Int	No	0	0
P88	DN	44	M	---	High	Yes	1	NA
P48.2	JAK2V617F$^-$	61	M	---	Int	No	0	0
P61	JAK2V617F$^-$	71	F	ASA, HU	Int	No	0	0
P65	JAK2V617F$^-$	50	F	---	Very low	No	0	0
P74	JAK2V617F$^-$	31	F	---	Very low	NA	0	1
P112	JAK2V617F$^-$	48	F	---	Very low	No	0	1

ASA: acetylsalicylic acid. CALR$^+$: positive for calreticulin mutation. DN: double negative for JAK2V617F and CALR mutation. F: female. HU: hydroxycarbamide (hydroxyurea). Int: intermediate. JAK2V617F$^+$: positive for JAK2V617F mutation. JAK2V617F$^-$: negative for JAK2V617F mutation. M: male. NA: not available. VE: previous vascular event.

Table S4. Demographic data and clinic-laboratory parameters from patients with primary myelofibrosis (PMF).

Code	Mutation	Age	Gender	Treatment	Prognostic score	VE	Fibrosis rate	Reticulin rate	Transfusion	Stage
P50	JAK2V617F^{+}	59	F	HU	DIPSS-PLUS 2	Yes	4	4	No	Fib (Post-PV)
P51	JAK2V617F^{+}	58	M	---	DIPSS-PLUS 3	Yes	4	4	Yes	Fib
P122	JAK2V617F^{+}	67	M	Ruxo	DIPSS-PLUS 4	No	3	3	No	Fib
P72	JAK2V617F^{+}	80	M	---	DIPSS-PLUS 1	No	3	3	No	P/P
P52	JAK2V617F^{+}	62	M	HU	DIPSS-PLUS 1	Yes	0	2	No	P/P
P97	JAK2V617F^{+}	73	M	---	DIPSS-PLUS 4	No	4	4	Yes	Fib
P103	JAK2V617F^{+}	79	M	---	DIPSS-PLUS 3	No	3	3	No	Fib
P54.2	JAK2V617F^{+}	68	M	HU	DIPSS-PLUS 3	No	2	2	No	Fib
P96	JAK2V617F^{+}	78	M	---	DIPSS-PLUS 4	No	3	3	Yes	Fib
P52.2	CALR^{+}	71	F	---	DIPSS-PLUS 4	No	3	3	No	Fib
P58	CALR^{+}	69	M	---	DIPSS-PLUS 3	No	3	3	Yes	P/P
P55.2	CALR^{+}	69	F	---	DIPSS-PLUS 2	Yes	2	2	No	P/P
P49.2	CALR^{+}	68	M	---	DIPSS-PLUS 2	No	2	2	No	P/P
P79	CALR^{+}	54	M	ASA, Anag	DIPSS-PLUS 1	Yes	3	3	No	P/P
P69	DN	73	M	---	DIPSS-PLUS 2	No	3	3	No	P/P
P113	DN	65	F	---	DIPSS-PLUS 4	No	4	4	Yes	Fib

Anag: anagrelide. ASA: acetylsalicylic acid. CALR^{+}: positive for calreticulin mutation. DN: double negative for JAK2V617F and CALR mutation. F: female. Fib: fibrotic. HU: hydroxycarbamide (hydroxyurea). Int: intermediate. JAK2V617F^{+}: positive for JAK2V617F mutation. JAK2V617F^{-}: negative for JAK2V617F mutation. M: male. NA: not available. P/P: prefibrotic/proliferative. Ruxo: ruxolitinib. VE: previous vascular event.

Table S5. Analysis of the soluble mediators levels in the bone marrow of healthy subjects (CTRL) and patients with polycythemia vera (PV), essential thrombocythemia (ET), and primary myelofibrosis (PMF).

Soluble mediator	Median (pg/mL) and range (min-max)				*p*-value					
	CTRL (n = 17)	PV (n = 19)	ET (n = 28)	PMF (n = 16)	PV x CTRL	PV x ET	PV x PMF	ET x CTRL	ET x PMF	PMF x CTRL
IL-6	9.46 (3.54 - 427.7)	15.6 (5.88 - 1887.96)	15.51 (6.4 - 3888)	9.1 (5.14 - 53.6)	0.1533	0.7353	0.0805	0.0841	**0.0195**	0.6630
IL-6Ra	43642 (30982 - 57312)	52108 (34688 - 68854)	50259 (20844 - 70846)	51576 (19672 - 70468)	**0.0005**	0.3245	0.6762	0.0613	0.5994	**0.0246**
IL-1β	46.10 (21.4 - 285.7)	63.08 (50.7 - 253.4)	63.08 (23.64 - 2850)	48.29 (28.4 - 146.3)	**0.0005**	0.4946	**0.0092**	**0.0073**	0.0603	0.5156
IL-10	35.2 (22.94 - 43.24)	42.5 (26.68 - 78.86)	33.14 (16.7 - 67.56)	31.21 (6.48 - 43.24)	0.0565	0.0577	**0.0037**	0.9123	0.2085	0.2192
IL-17a	24.7 (15.08 - 94.36)	35.66 (26.6 - 67.7)	29.88 (11.08 - 135.3)	26.6 (13.68 - 43.04)	**<0.0001**	**0.0179**	**0.0033**	**0.0144**	0.1175	0.4049
IL-12p70	382.3 (186-7 - 1121)	452.42 (263.24 - 781)	427.1 (180.3 - 725.7)	369 (206.7 - 1065)	**0.0188**	0.2735	**0.0279**	0.0879	0.0953	0.8798
IL-18	191.9 (98.22 - 408.3)	349.86 (173.1 - 1405)	252.5 (138.5 - 2172)	350.1 (161.6 - 884)	**<0.0001**	0.0899	0.8895	**0.0042**	0.1204	**0.0001**
CXCL8	36.94 (13.94 - 156.2)	98.26 (23.96 - 3788)	192.4 (14.44 - 7464)	71.73 (25.9 - 3118)	**0.0198**	0.5802	0.5287	**0.0046**	0.1837	**0.0379**

CXCL10	65.44 (28.7 - 105.8)	276.7 (19.34 - 2674)	79.74 (26.8 - 1882)	93.12 (60.06 - 981.5)	**0.0040**	**0.0429**	0.0634	**0.0490**	0.1969	**0.0027**
CXCL12	967.7 (631 - 1488)	1111.4 (933.2 - 2170)	1047 (390.3 - 2134)	985.1 (441.9 - 1649)	**0.0102**	0.0941	**0.0153**	0.3556	0.4352	>0.9999
CCL2	288.9 (78 - 459.2)	491.86 (247.5 - 8140)	403.2 (111 - 6180)	372.2 93.62 - 781.8)	**0.0004**	0.3519	**0.0432**	**0.0038**	0.4144	0.0643
CCL5	17650 (8164 - 20632)	19716 (16646 - 21328)	19877 (4480 - 21042)	18892 (8306 - 20374)	**0.0007**	0.4951	**0.0164**	**0.0015**	**0.0103**	0.3598
VEGF	397.1 (267.6 - 579.3)	664.58 (397.1 - 1522)	518.3 (203.3 - 1254)	393.7 (249.8 - 2684)	**<0.0001**	0.0673	**0.0025**	**<0.0001**	**0.0202**	0.4483
VEGF-R2	9212 3646 - 12766)	11128 7000 - 23278)	10479 (5052 - 17688)	9373 (3906 - 17092)	**0.0331**	0.5437	0.1739	0.1003	0.3546	0.5510
TNF-α	44.9 (16.44 - 76.7)	56.28 (39.84 - 193.8)	54.3 (23.66 - 242.2)	48.52 (19.42 - 111.9)	**<0.0001**	0.2082	**0.0107**	**0.0096**	0.2665	0.1904
IFN-γ	1855 (982-7 - 2164)	2164 (1953 - 2358)	2092 (538.9 - 2358)	2022 (1089 - 2240)	**<0.0001**	**0.0364**	**0.0010**	**0.0003**	0.0865	0.2258
GM-CSF	35.3 (17.68 - 70.82)	50.14 (31.78 - 112.6)	48.18 (21.08 - 140.8)	38.04 (21.68 - 78.54)	**<0.0001**	0.2690	**0.0007**	**0.0262**	0.1296	0.3215
M-CSF	6116 (3654 - 7776)	6778 (6012 - 8922)	6494 (2638 - 10592)	6012 (3782 - 7776)	**0.0071**	0.1745	**0.0056**	0.3029	0.2222	0.5991
G-CSF	569.3 (479.5 - 21292)	653.12 (504.9 - 1038)	584.2 (390.3 - 920.7)	594.3 (471.4 - 859.6)	0.0731	**0.0046**	**0.0231**	0.6465	0.8420	0.6753

HGF	392.7 (178.3 - 780.4)	793.88 (373 - 5888)	396.3 (169.4 - 975.5)	342.4 (158.1 - 5138)	**<0.0001**	**0.0003**	**0.0060**	0.3869	0.8327	0.9929

Bold values indicate statistical difference ($p < 0.05$)

Table S6. Results of the correlation analysis between soluble mediator levels and hematological parameters from patients with polycythemia vera (n=19).

Soluble mediator	Comparison (*p*-value, *r*- value)				
	HGB	HCT	WBC	RBC	PLT
IL-6	0.4659, 0.0211	0.4631, -0.0228	0.4461, 0.1474	0.4957, 0.0026	0.4461, -0.0333
IL-6Ra	0.2033, 0.2021	**0.0452**, 0.3993	**0.0255**, 0.4537	**0.0073**, 0.5505	0.2272, 0.1825
IL-1β	0.2418, -0.1711	0.3779, -0.0765	0.3943, 0.0659	0.2853, 0.1389	0.3029, 0.1265
IL-10	0.2176, -0.1903	0.0993, -0.3086	0.2959, -0.1314	0.3685, -0.0825	0.1352, -0.2663
IL-17a	0.4978, 0.0013	0.3867, -0.0708	0.2165, 0.1912	0.0918, 0.3187	0.0581, 0.3726
IL-12p70	0.0728, -0.3470	0.0637, -0.3624	0.3078, 0.1231	0.1132, 0.2912	0.4971, -0.0018
IL-18	0.1580, -0.2431	0.1933, -0.2107	0.1079, -0.2976	0.4460, -0.0334	0.1243, -0.2783
CXCL8	0.4208, 0.0492	0.4744, 0.0158	0.3262, 0.1105	0.2795, 0.1430	0.2230, -0.1860
CXCL10	0.2281, -0.1818	0.3108, -0.1211	0.4489, -0.0316	0.3352, 0.1044	**0.0113**, -0.5193
CXCL12	0.4179, -0.0510	0.2384, -0.1738	0.1785, -0.2238	0.4159, -0.0522	0.4278, 0.0448
CCL2	0.4730, 0.0167	0.3366, -0.1035	0.2430, -0.1702	0.1718, -0.2299	**0.0190**, -0.4789
CCL5	**0.0191**, -0.4787	**0.0007**, -0.6772	**0.0319**, -0.4333	0.1756, -0.2264	0.4773, 0.0140
VEGF	**0.0493**, 0.3902	0.1260, 0.2764	**0.0337**, 0.4283	0.0546, 0.3793	0.0857, 0.3273

VEGF-R2	0.0990, -0.3090	0.1981, -0.2066	0.1695, -0.2321	0.3021, -0.1271	0.2853, -0.1389
TNF-α	0.3994, 0.0627	0.4643, 0.0201	0.0516, 0.3854	**0.0178**, 0.4843	0.1134, 0.2910
IFN-γ	**0.0454**, -0.3988	**0.0066**, -0.5572	0.1060, -0.3000	0.1756, -0.2265	0.1483, 0.2527
GM-CSF	0.3463, -0.0971	0.2005, -0.2045	0.2947, -0.1322	0.4864, -0.0084	0.0598, -0.3693
M-CSF	**0.0262**, -0.0555	**0.0067**, -0.5557	0.3426, -0.0995	0.4107, -0;0555	0.4615, -0.02378
G-CSF	0.2509, -0.1642	0.1375, -0.2639	0.3807, 0.0746	0.1287, 0.2734	0.0896, 0.3217
HGF	0.2337, 0.1774	0.3794, 0.0754	0.1207, 0.2825	**0.0124**, 0.5124	0.0992, 0.3088

Bold values indicate statistical difference ($p < 0.05$). HCT: hematocrit. HGB: hemoglobin concentration. RBC: red blood cell count. PLT: platelet count. WBC: white blood cell count.

Table S7. Results of the correlation analysis between soluble mediator levels and hematological parameters from patients with essential thrombocythemia (n=28).

Soluble mediator	Comparison (*p*-value, *r*- value)				
	HGB	HCT	WBC	RBC	PLT
IL-6	0.4749, 0.0125	0.3835, 0.0586	0.3035, -0.1016	0.3320, -0.0859	0.2256, -0.1484
IL-6Ra	**0.0266**, 0.3691	0.1350, 0.2158	**0.0083**, 0.4489	**0.0144**, 0.4133	0.1405, 0.2111
IL-1β	**0.0288**, 0.3630	**0.0402**, 0.3360	0.4370, 0.0314	0.1735, 0.1846	0.4124, 0.0438
IL-10	**0.0371**, 0.3428	0.0893, 0.2616	0.4779, -0.0110	0.3786, 0.0612	0.2903, -0.1091
IL-17a	0.0870, 0.2644	0.1333, 0.2173	0.1758, 0.1829	0.4305, 0.0347	0.1361, 0.2149
IL-12p70	0.1000, 0.2497	0.1967, 0.1678	0.3635, 0.0690	0.2226, 0.1503	0.1922, 0.1710
IL-18	**0.0204**, 0.3889	0.0758, 0.2782	0.2504, 0.1327	0.4035, 0.0484	0.2106, -0.1583
CXCL8	0.4724, 0.0137	0.2602, 0.1268	0.4017, -0.0493	0.4901, -0.0049	0.4233, 0.0383

CXCL10	0.2378, 0.1405	0.1426, 0.2092	0.2903, 0.1091	0.4928, -0.0036	0.2721, -0.1196
CXCL12	0.4732, 0.0133	0.4045, -0.0478	0.3552, -0.0734	0.3708, -0.0652	0.4053, -0.0244
CCL2	0.3938, 0.0533	0.1894, 0.1730	0,4393, -0.0303	0.3795, -0.0507	0.2014, -0.1645
CCL5	0.3187, 0.0931	0.3683, 0.6665	0.0858, 0.2658	0.3265, 0.0889	**0.0477**, 0.3213
VEGF	0.2122, 0.1572	0.2273, 0.1472	0.4,48, -0.0325	0.4090, 40.0455	0.3850, 0.0578
VEGF-R2	0.4829, -0.0085	0.3596, -0.0711	0.0744 0.2801	**0.0476**, 0.3215	**0.0179**, 0.3985
TNF-α	0.0675, 0.2895	0.2859, 0.1116	0.3024, -0.1022	0.1743, 0.1840	0.2780, -0.1162
IFN-γ	0.1505, 0.2026	0.2996, 0.1038	0.3587, 0.0716	0.4900, -0.0050	0.1678, 0.1889
GM-CSF	0.0527, 0.3126	**0.0182**, 0.3973	0.4592, 0.0203	0.0861, 0.2655	0.3984, 0.0510
M-CSF	0.2064, 0.1611	0.4583, 0.0207	0.4470, 0.0264	0.4295, 0.0352	0.4078, 0.0461
G-CSF	**0.0345**, 0.3486	0.1214, 0.2282	0.0992, 0.2506	0.2180, 0.1533	0.2083, 0.1598
HGF	0.2663, 0.1231	0.3970, 0.0517	0.1060, 0.2434	0.4614, -0.0192	0.1869, 0.1748

Bold values indicate statistical difference ($p < 0.05$). HCT: hematocrit. HGB: hemoglobin concentration. RBC: red blood cell count. PLT: platelet count. WBC: white blood cell count

Table S8. Results of the correlation analysis between soluble mediator levels and hematological parameters from patients with primary myelofibrosis (n=16).

Soluble mediator	Comparison (*p*-value, *r*- value)				
	HGB	HCT	WBC	RBC	PLT
IL-6	**0.0150**, -0.5453	**0.0364**, -0.1252	0.3191, -0.1252	**0.0270**, -0.4908	0.0879, -0.4011
IL-6Ra	0.4132, -0.0597	0.4462, -0.0368	0.3028, 0.1398	0.4376, -0.0427	0.2436, 0.2118

IL-1β	0.4978, 0.0015	0.4439, -0.0384	0.2705, -0.1652	0.4225, 0.0531	0.3943, 0.0825
IL-10	**0.0119**, -0.4798	**0.0114**, -0.4832	0.3517, -0.0860	**0.0152**, -0.4619	0.1550, -0.2460
IL-17a	0.1101, -0.3165	0.1228, -0.3082	0.2732, 0.1630	0.0968, -0.3336	0.3557, -0.1137
IL-12p70	0.2536, -0.1790	0.2030, -0.2232	0.3454, 0.1079	0.0901, -0.3528	0.1710, 0.2869
IL-18	0.2541, -0.1786	0.3357, -0.1150	0.6340, 0.0944	0.1144, -0.3188	0.2182, -0,2366
CXCL8	0.1256, -0.3046	0.1933, -0.2324	0.3685, 0.0912	0.2253, -0.2031	0.2468, -0.2088
CXCL10	**0.0028**, -0.6583	**0.0034,** -0.6461	0.1854, -0.2399	**0.0226**, -0.5066	0.0872, -0.4011
CXCL12	0.2034, 0.2229	0.1774, 0.2478	0.3518, 0.1032	0.2230, 0.2052	0.2350, 0.2201
CCL2	0.1393, -0.2884	0.1064, -0.3294	0.3010, -0.1412	0.2820, -0.1560	0.2706, 0.1868
CCL5	0.3974, 0.0706	0.3401, 0.1118	0.2255, 0.2029	0.3808, 0.0824	0.0840, 0.4066
VEGF	0.4098, -0.0620	0.4311, -0.0472	0.4870, 0.0089	0.1477, -0.2790	0.2210, 0.2338
VEGF-R2	0.3183, 0.1280	0.2058, 0.2206	**0.0349**, 0.4647	0.2691, 0.1663	0.1274, 0.3407
TNF-α	0.3200, -0,1268	0.4079, -0.0633	0.2969, 0.1443	0.1843, 0.2410	0.2642, 0.1926
IFN-γ	0.4913, 0.0059	0.4718, 0.0193	0.1299, 0.2995	0.4956, -0.0030	0.1009, 0.3789
GM-CSF	**0.0014**, -0.6934	**0.0029**, -0.6568	0.4058, 0.0648	**0.0120**, -0.5601	0.4573, -0.0033
M-CSF	0.3743, 0.0870	0.3102, 0.1341	0.0682, 0.3891	0.4247, 0.0516	0.3770, 0.0964
G-CSF	0.2650, -0.1696	0.2817, -0.1562	0.4935, 0.0044	0.1275, -0.3024	0.4432, -0.0441
HGF	0.4849, -0.0103	0.3705, -0.0898	0.2009, 0.2252	0.1261, -0.3041	0.2613, 0.1953

Bold values indicate statistical difference ($p < 0.05$). HCT: hematocrit. HGB: hemoglobin concentration. RBC: red blood cell count. PLT: platelet count. WBC: white blood cell count

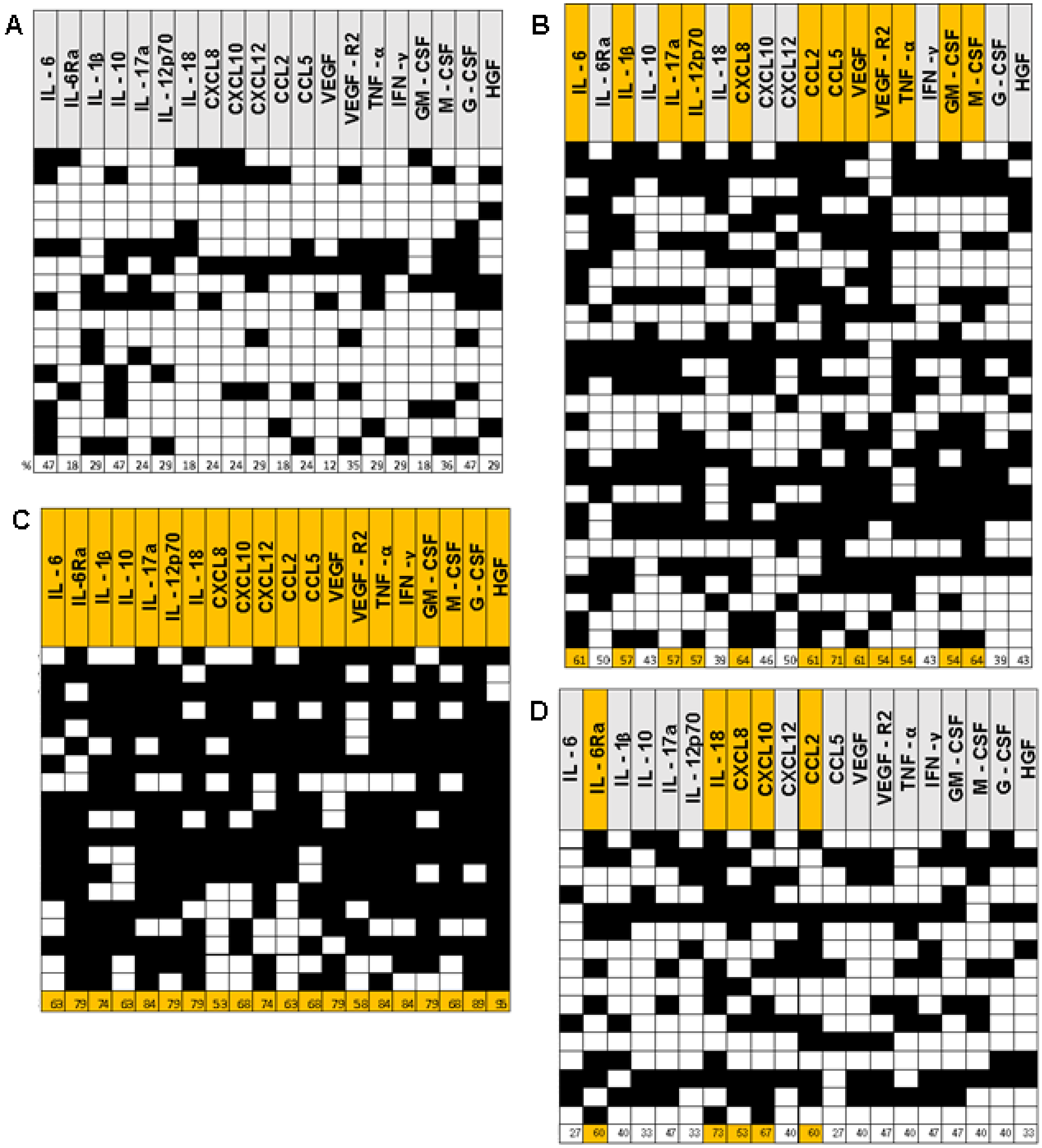

Figure S1. Bone marrow soluble mediator signatures. **(A)** Healthy subjects (CTRL, n=17). CTRL group showed low frequency (<50% of group individuals) of high soluble mediator producers for all analyzed mediators. Patients with **(B)** essential thrombocythemia (ET, n=28), **(C)** polycythemia vera (PV, n=19), and **(D)** primary myelofibrosis (PMF, n=16). Soluble mediator production was stratified as high and low. Black blocks indicate high producers and white blocks indicate low producers of each soluble mediator analyzed. Each column represents one analyte, and each row represents one subject. The number below each column corresponds to the frequency of high producers (%). The soluble mediator is highlighted when the frequency of high producers exceeded 50% of the group individuals, and the soluble mediator was considered relevant for the disease.

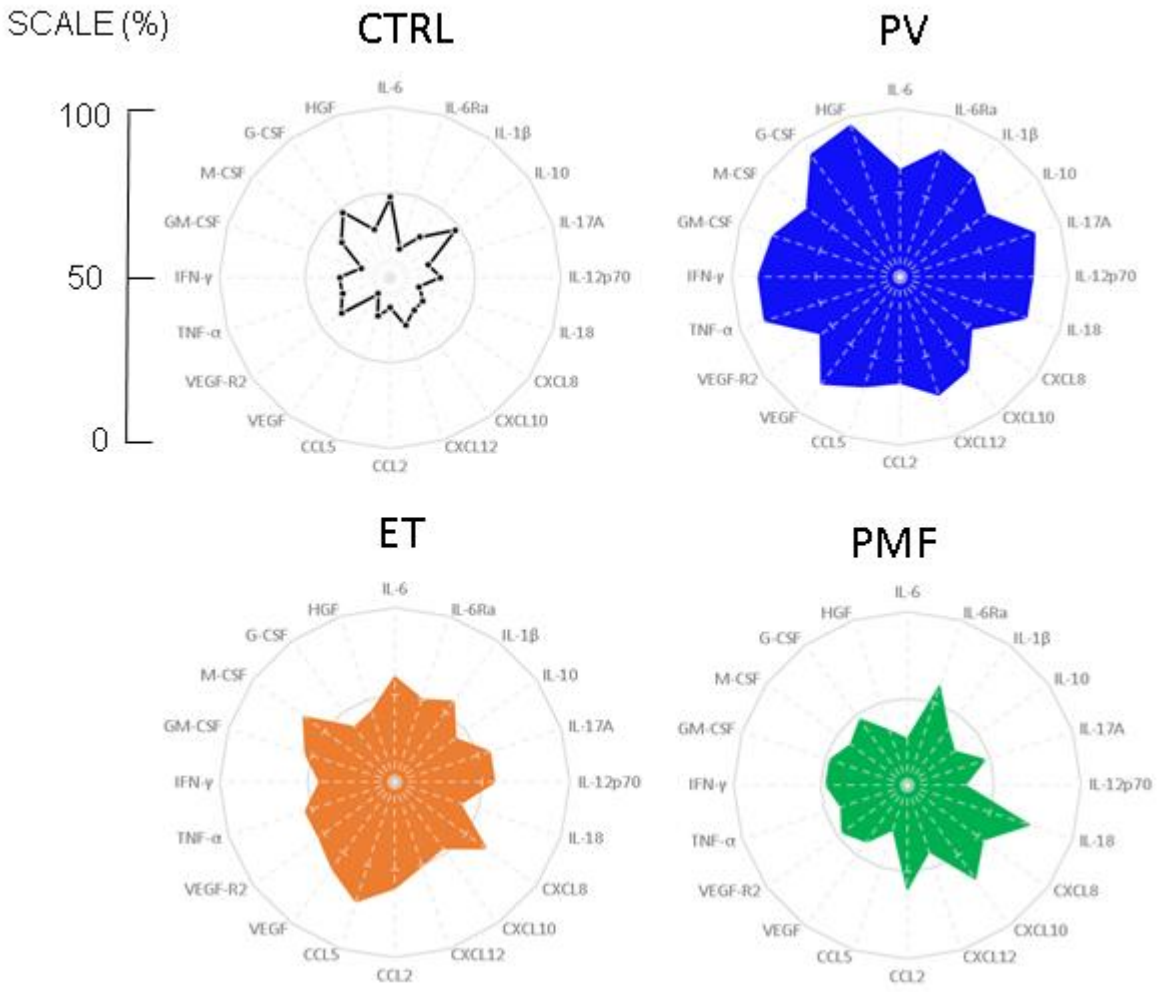

Figure S2. Spider charts of soluble mediator signatures from healthy subjects (CTRL, n=17) and patients with essential thrombocythemia (ET, n=28), polycythemia vera (PV, n=19), and primary myelofibrosis (PMF, n=16). Comparison of frequency of overall mediator production among the disease subtypes. The results are expressed in percentage.

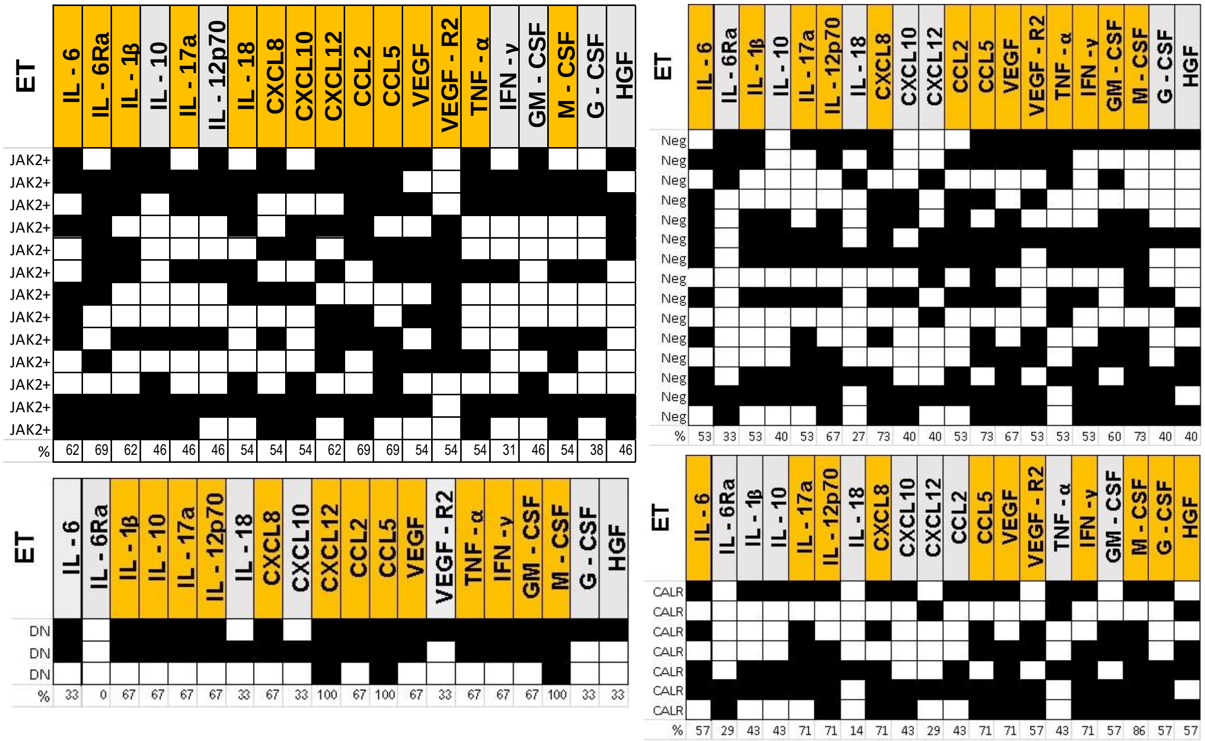

Figure S3. Association between driver mutation *statu*s and soluble mediator signature in patients with essential thrombocythemia (ET). JAK2V617^{+} (n=13; ET patients positive for JAK2V617F mutation), JAK2V617F^{-} (n=15; ET patients negative for JAK2V617F mutation), CALR^{+} (n=7; patients positive for calreticulin mutation), DN (n=3; double negative patients). Soluble mediator production was stratified as high and low. Black blocks indicate high producers and white blocks indicate low producers of each soluble mediator analyzed. Each column represents one analyte, and each row represents one individual. The number below each column corresponds to the frequency of high producers. Yellow boxes indicate that more than 50% of the individuals of the group were high producers of that soluble mediator.

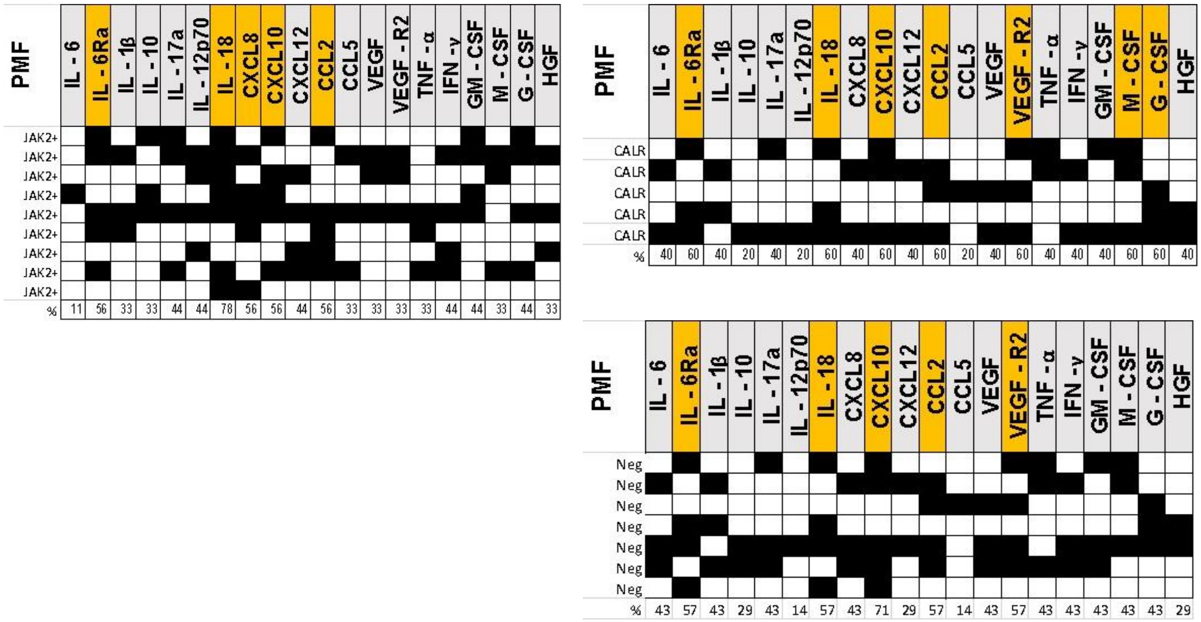

Figure S4. Association between driver mutation status and soluble mediator signature in patients with primary myelofibrosis (PMF). JAK2V617^{+} (n=9; PMF patients positive for JAK2V617F mutation), JAK2V617F^{-} (n=7; PMF patients negative for JAK2V617F mutation), CALR^{+} (n=5; PMF patients positive for calreticulin mutation). Soluble mediator production was stratified as high and low. Black blocks indicate high producers and white blocks indicate low producers of each soluble mediator analyzed. Each column represents one analyte, and each row represents one individual. The number below each column corresponds to the frequency of high producers. Yellow boxes indicate that more than 50% of the individuals of the group were high producers of that soluble mediator.
